# Achieving consensus on the language of obesity: a modified Delphi study

**DOI:** 10.1016/j.eclinm.2023.102061

**Published:** 2023-07-27

**Authors:** Cecilie Heerdegen Jepsen, Jacqueline Bowman-Busato, Thomas Allvin, Niamh Arthurs, Gijs H. Goossens, Elisabeth Govers, Benjamin Assad Jaghutriz, Vicki Mooney, Bart Van der Schueren, Carel W. le Roux

**Affiliations:** a Novo Nordisk A/S, Søborg, Denmark; b European Association for the Study of Obesity (EASO), Brussels, Belgium; c European Federation of Pharmaceutical Industries and Associations, Brussels, Belgium; d European Association for the Study of Obesity (EASO), Dublin, Ireland; e Department of Human Biology, NUTRIM School of Nutrition and Translational Research in Metabolism, Maastricht University Medical Center+, Maastricht, the Netherlands; f European Federation of Associations of Dietitians (EFAD), Amsterdam, the Netherlands; g Lilly Deutschland GmbH, Medical Department, Bad Homburg vor der Höhe, Germany; h The European Coalition for People Living with Obesity (EASO ECPO), Dublin, Ireland; i Department of Endocrinology, University Hospitals Leuven, Leuven, Belgium; j Laboratory of Clinical and Experimental Endocrinology, University of Leuven, Leuven, Belgium; k Diabetes Complications Research Centre, University College Dublin, Dublin, Ireland

**Keywords:** Obesity, Overweight, Language, Chronic disease, Noncommunicable disease, Pre-obesity taxonomy

## Abstract

**Background:**

Obesity is recognized by the World Health Organization as a chronic disease. As such, it should be referred to using the language of chronic diseases, with correct and established terminology and definitions. This study was designed to map the current language used to discuss obesity and to compare this with the standard language used for chronic disease.

**Methods:**

We performed a modified Delphi study to identify the language of chronic disease that is being used in the context of obesity, and to identify discrepancies and potential use of inadequate language with respect to the standard language used for chronic diseases. Participants (*n* = 24) were identified from relevant stakeholder groups and desk research, and included patients, healthcare professionals, policymakers, researchers, industry, and payers (social insurers) of 18 nationalities/regions in Europe, North/South America, and South Africa. Participants were enrolled between 20.10.2020 and 30.10.2020. The study comprised two rounds of qualitative surveys. In Round 1, participants responded to six open-ended questions. Round 2 comprised 38 statements based on key terms/themes identified in Round 1 and covered the definition, causes, progression, treatment, management, and complications of obesity. Consensus was defined as ≥70% participant agreement on a statement.

**Findings:**

All participants completed Round 1 and 23 participants completed Round 2. In Round 2, consensus was reached for 28 of the 38 statements. Participants reached a consensus regarding the use of statements that acknowledge the heterogeneous nature of obesity, but not on the use of statements that: defined obesity based on body mass index; regarded psychological, physical, or physiological factors among the main causes of obesity; or implied that weight loss should be the aim of obesity treatment.

**Interpretation:**

This study uses expert consensus to provide insight into the language used to describe obesity as a chronic disease, and forms the basis for a unified language of obesity.

**Funding:**

10.13039/501100010767Innovative Medicines Initiative, Novo Nordisk A/S.

**Research in context**

**Evidence before this study**

We conducted a literature review to examine the current language of obesity, in order to identify barriers and facilitators for key stakeholder groups in discussing obesity as a chronic, relapsing disease. We searched PubMed, CINHAL, EBSCO, Google Scholar, and Web of Science to identify all relevant articles covering the language used to discuss obesity published from inception to July 2021. The following search methodology was adopted: PubMed: (Needs) AND (challenges) AND (obesity risk) AND (further complications); CINAHL: (predicting) AND (response) AND (obesity) AND (treatment); EBSCO: (predicting) AND (response) AND (obesity) AND (treatment); Google Scholar: (Needs) AND (challenges) AND (‘predictor OR predictors’) AND (obesity) AND (‘complications’ OR ‘risk’); and Web of Science: ‘stakeholder’ AND ‘challenges’ AND ‘addressing’ AND ‘obesity’ AND ‘chronic relapsing disease’. The search was widened to include “stakeholder” AND “challenges” OR “barriers” OR “obstacles” AND “addressing” OR “managing” OR “treating” AND “obesity”. The references cited within relevant articles were also examined, and where available we utilised the "related articles/citations" function. Relevant policies and guidance documents from regulators were also sourced and reviewed. Inclusion criteria included free full texts available in the English language; however, we ensured a broad representation of countries and cultures. Examination of our literature review confirmed that the appropriate terminology was not being adopted in the correct context, and that there was a lack of language pertaining to chronic disease when discussing obesity.

**Added value of this study**

Using the modified Delphi method, expert representatives of different stakeholder groups took part in a taxonomic exercise with the aim of mapping the language that is currently used to discuss obesity as a chronic, relapsing disease. We concluded that there is consensus (i.e. ≥70% of participant agreement) around the use of several key statements that refer to obesity as a chronic, relapsing disease with many root causes as well as factors that affect its progression, including genetic, environmental, and (epi)genetic factors. The results also highlighted discrepancies compared with the established language of chronic diseases, and where consensus was still needed with respect to the language and context in some aspects of obesity. Notably, the statements that did not achieve consensus (i.e. <70% of participant agreement) around their use, included notions around the balance between energy intake and expenditure as a cause or exacerbating factor of obesity, or body mass index as a diagnostic biomarker for obesity. The results of this study support a holistic approach to obesity treatment and management and demonstrate strong consensus on the broad range of diseases that can be considered complications of obesity.

**Implications of all the available evidence**

The present study demonstrates a strong consensus from stakeholders on the use of statements that identify obesity as a chronic, relapsing disease with various causes and factors that affect disease progression. The consensus on the language of obesity shown by this study is important to understand where we still need to develop a more nuanced unified language, which takes the complexity of the disease into consideration. Ultimately, this may result in overcoming the barriers and challenges that surround treatment and management of people living with obesity.

## Introduction

The World Health Organization has recognised obesity as a disease since 1948,^1^ and in 2021, the European Commission categorised obesity as a chronic disease.^2^ Accordingly, obesity should be referred to using the language of chronic diseases, with correct and established terminology and definitions.^3^ The language of chronic diseases is usually precise, accurate, and respectful. However, despite the international recognition of obesity as a chronic disease, the language used to describe obesity is often inaccurate and sometimes not sufficiently respectful, which can contribute to the misunderstanding of obesity, encourage weight stigma, and lead to systemic bias.^4^, ^5^, ^6^ This may create barriers and challenges to the treatment and management of people living with obesity.

The World Obesity Federation has highlighted how the inconsistency in terminology used to discuss obesity can reinforce misconceptions and incorrectly simplify the aetiology and drivers of disease progression.^7^ In addition, the language used for obesity is not aligned with the language that is used for chronic disease. Therefore, there is an unmet need to investigate the current language of obesity among relevant stakeholders, with the aim of identifying areas of conflict and/or language inadequacy.

The Innovative Medicines Initiative Stratification of Obesity Phenotypes to Optimise Future Therapy (IMI-SOPHIA) one voice exercise was conducted to map the current language of obesity and to evaluate this against the standard language used for chronic disease. It is intended to help optimise treatment by building a framework to align language accuracy, precision, and respectfulness with regards to obesity. More broadly, the goal of the SOPHIA project is to change the narrative around obesity, to make it patient-centric and equitable and to underline that it is a chronic disease. It should be noted that review and discussion of weight stigma was outside the scope of the current study.

## Methods

### Study design

**Fig. 1:**
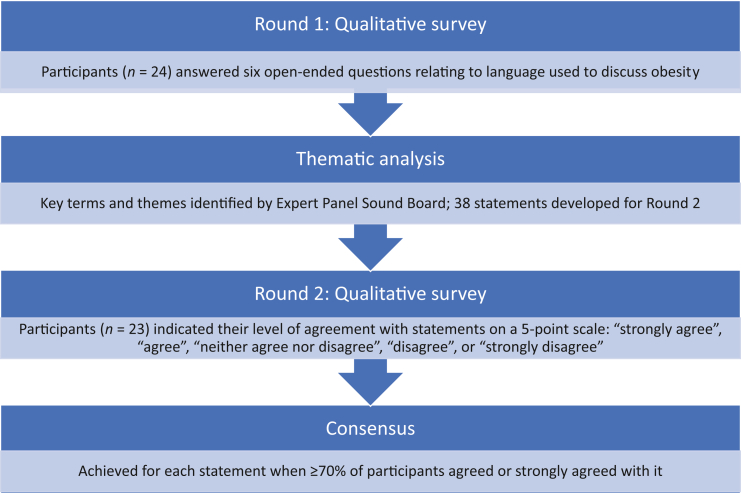
Flow chart of the modified Delphi process.

The study involved a modified Delphi method to map current consensus among experts on the language (words, phrasing, and usage) and scope (definition, causes, exacerbating factors, treatment options, management, and medical complications) around the language we use when discussing obesity as a chronic disease (Fig. 1). The CREDES checklist was used to ensure adherence to EQUATOR reporting guidelines.

The Delphi method is recommended as a reliable means of determining consensus for a defined clinical problem.^8^, ^9^, ^10^, ^11^ The method involves an iterative process of repeated rounds of voting and is effective for determining expert group consensus and where opinion is important.^12^ The classical Delphi method starts with unstructured gathering of opinions, while the modified method initially gathers responses to a fixed set of questions.^13^ Studies have demonstrated that the modified Delphi method can be superior to the original Delphi method and perceived as highly effective.^14^^,^^15^ In this study, the modified Delphi method consisted of two rounds of qualitative surveys delivered through an online platform. This exercise was supported by an Expert Panel Sounding Board representative of different stakeholder sectors to ensure clarity, minimise bias in the interpretation of the questions and statements, and monitor the process.

Round 1 consisted of six open-ended questions, which had an open-comment field answer box for responses: 1) Do you have a definition of obesity that is used in your environments and/or professional organisation?; 2) What would you say are the causes of obesity?; 3) What would you say are the factors that exacerbate obesity at individual level?; 4) What do you consider is meant by ‘obesity treatment’?; 5) What do you consider is meant by ‘obesity management’?; and 6) What do you consider to be the main medical complications of obesity?

The questions reflected European Association for the Study of Obesity position statement themes, and were also informed by our literature review findings, whereby obesity was primarily defined by weight or body mass index (BMI).^16^^,^^17^ When obesity is inadequately understood, it can result in poor treatment or lack of treatment and a multitude of obesity complications can arise; subsequently presenting far greater barriers to its treatment and management.^18^ We also found a paucity of evidence in the literature for ways of combining multiple factors to predict individual responses to obesity treatments, thereby improving obesity management.^19^, ^20^, ^21^, ^22^

The Expert Panel Sounding Board discussed the findings after each question in Round 1 and worked with the core research team to develop the statements for Round 2. Round 2 comprised 38 statements based on key terms and themes identified in Round 1, divided into six sections covering: 1) the definition of obesity; 2) the causes of obesity development; 3) the disease progression of obesity; 4) the treatment of obesity; 5) the management of obesity; and 6) the complications of obesity. Participants were asked to indicate their level of agreement with the statements: ‘strongly agree’, ‘agree’, ‘neither agree nor disagree’, ‘disagree’, or ‘strongly disagree’. After completing sections one to six, the participants were asked to rank the statements according to how suitable they found them to include in the definition/common vocabulary when referring to each section.

All responses were anonymised and the Delphi exercise was conducted in English. A statement generated through the Delphi exercise was said to have achieved consensus when ≥70% of participants either agreed or strongly agreed with its use. There is no rule that specifies which cut-off value for consensus should be applied using the Delphi technique, but commonly applied levels vary between 51% and 80%.^13^

### Participants

Expert participants were recruited from the IMI-SOPHIA work package 8 task force, as well as a wider field, to take part in the Delphi exercise. Steps in the selection process included identifying relevant stakeholder groups, personally contacting individuals from within these groups and letting them nominate others for inclusion, and performing desk research to identify any other key opinion leaders. Chosen participants represented several stakeholder groups: patients, healthcare professionals, policymakers, researchers, industry, and payers (social insurers). A similar process was used to recruit the Expert Panel Sounding Board. Ethics committee approval was not required for this study. All participants provided written informed consent prior to enrolment.

### Data analysis

A thematic analysis was conducted for responses to questions in Round 1, with responses analysed and grouped into broad overarching themes. Once key terms and themes had been identified, they were quantified by how many times they appeared in the responses. The top five mentioned themes, and/or those mentioned six times or more, were used as a base to produce statements which were included in Round 2.

### Role of the funding source

The funder of the study had no role in study design, data collection, data analysis, data interpretation, or writing of the report. All authors had access to the dataset and had final responsibility for the decision to submit for publication.

## Results

Of 56 individuals invited to participate in the Delphi process, 24 (43%) accepted the invitation. All participants completed Round 1 (100% response rate) and 23 participants completed Round 2 (95·8% response rate; Table 1). Fifty-eight percent of participants identified as female. Participants identified as being of 18 nationalities/regions: American, Brazilian–Italian, British, Canadian, Catalan (Spanish), Czech, Danish, Dutch, German, Greek, Italian, Portuguese, Romanian, Slovenian, South African, Spanish, Swedish, and Turkish (Table 1).

**Table 1:** Demographic characteristics of the participants.

	No. of participants recruited (*n* = 24)	Round 1^a^ (*n* = 24)	Round 2^b^ (*n* = 23)
**Gender**			
Male	8	NA	NA
Female	14	NA	NA
Agender	1	NA	NA
Unanswered	1	NA	NA
**Nationality/region**		NA	NA
American	1	NA	NA
Brazilian–Italian	1	NA	NA
British	1	NA	NA
Canadian	2	NA	NA
Catalan (Spanish)	1	NA	NA
Czech	1	NA	NA
Danish	1	NA	NA
Dutch	1	NA	NA
German	1	NA	NA
Greek	1	NA	NA
Italian	3	NA	NA
Portuguese	1	NA	NA
Romanian	2	NA	NA
Slovenian	1	NA	NA
South African	1	NA	NA
Spanish	1	NA	NA
Swedish	1	NA	NA
Turkish	1	NA	NA
Unanswered	2	NA	NA
**Role**			
Healthcare professional	4	4	4
Industry	4	4	4
Patient	6	6	5
Payers (social insurers)	3	3	3
Policymakers	3	3	3
Researchers	4	4	4

a Number of participants who responded in Round 1.

b Number of participants who responded in Round 2.

The most common responses in Round 1 are shown in Supplementary Figures S1 and S2. In Round 2, consensus (≥70% of participants agreed or strongly agreed) was reached for 28 of the 38 statements (Table 2). In terms of definitions of obesity, consensus was achieved for the use of the statements: ‘Obesity is a chronic, lifelong disease’; ‘Obesity is a complex disease’; and ‘Obesity is defined as abnormal or excessive fat accumulation that may impair, or present a risk to, health’. Consensus was not reached (i.e., <70% participants agreed or strongly agreed) around the idea that obesity can be measured by calculating BMI. Consensus was achieved for the use of statements that indicated that the main causes of obesity development include genetic factors, environmental factors, or the interaction between these two. The idea that an imbalance between energy intake and energy expenditure is among the main causes of developing or exacerbating obesity did not achieve consensus. For disease progression, consensus was achieved for the use of statements that indicated that the main factors that exacerbate obesity include biological predisposition, socioeconomic, psychological, and environmental factors, and weight stigma, bias, and lack of social support. Consensus was achieved for the use of statements that indicated that obesity treatment options should take a multidisciplinary and holistic approach, be person-centred and individualised, and aim at improving health outcomes. Consensus was achieved for the use of statements that indicated that obesity management should refer to the lifelong approach to obesity as a chronic, relapsing disease, that obesity management should include a personalised, holistic plan, co-designed by the healthcare team and the individual, and that it should aim to improve overall health and quality of life, and not only focus on weight management. All statements regarding complications of obesity reached consensus.

**Table 2:** Statements with or without consensus among participants.

Statement	Consensus reached^a^
**Definitions of obesity**	
Obesity is a chronic, lifelong disease	Yes
Obesity is a complex disease	Yes
Obesity is defined as abnormal or excessive fat accumulation that may impair, or present a risk to, health	Yes
**Obesity is defined as abnormal or excessive adiposity that may impair, or present a risk to, health**	**No**
**Obesity is a chronic, complex disease defined by excessive adiposity that may impair health**	**No**
**Obesity can be measured by calculating body mass index**	**No**
**Causes of obesity development**	
Genetic factors are among the main causes of developing obesity	Yes
Environmental factors are among the main causes of developing obesity	Yes
**Psychological factors are among the main causes of developing obesity**	**No**
**Pre-existing medical conditions are among the main causes of developing obesity**	**No**
**Endocrine disorders are among the main causes of developing obesity**	**No**
**Medication is among the main causes of developing obesity**	**No**
The interaction between genetics and environmental factors is among the main causes of developing obesity	Yes
**An imbalance between energy intake and energy output is among the main causes of developing obesity**	**No**
**Factors exacerbating obesity**	
Psychological factors are among the main factors that exacerbate obesity	Yes
Biological (including genetic) predisposition is among the main factors that exacerbate obesity	Yes
Environmental factors are among the main factors that exacerbate obesity	Yes
**An imbalance between energy intake and energy output is among the main factors that exacerbate obesity**	**No**
Stigma, bias, and lack of social support are among the main factors that exacerbate obesity	Yes
Socioeconomic factors are among the main factors that exacerbate obesity	Yes
**Obesity treatment options**	
Obesity treatment options should include bariatric surgery, where appropriate	Yes
Obesity treatment options should include pharmacological solutions, where appropriate	Yes
Obesity treatment options should include lifestyle interventions	Yes
Obesity treatment options should include psychological therapies, where appropriate	Yes
Obesity treatment options should take a multidisciplinary and holistic approach	Yes
Obesity treatment options should be person-centred and individualised	Yes
Obesity treatment options should aim at improving health outcomes	Yes
**Obesity treatment options should aim at weight loss**	**No**
**Obesity management**	
Obesity management should refer to the lifelong approach to obesity as a chronic, relapsing disease	Yes
Obesity management should include a personalised, holistic plan	Yes
An obesity management plan should be co-designed by the healthcare team and the individual	Yes
Obesity management should aim to improve overall health and quality of life	Yes
Obesity management should not only focus on weight management	Yes
**Medical complications of obesity**	
Cardiovascular diseases (such as hypertension or heart disease) are among the main medical complications of obesity	Yes
Type 2 diabetes is among the main medical complications of obesity	Yes
Certain cancers are among the main medical complications of obesity	Yes
Mental health conditions (such as anxiety and depression) are among the main medical complications of obesity	Yes
Mechanical complications (such as arthritis and obstructive sleep apnoea) are among the main medical complications of obesity	Yes

a Consensus was reached if agreed by ≥70% of participants.

Participants provided detailed comments on the statements from each section (Table 3). The free-text comments from participants emphasised the heterogeneity of language used around obesity. Further, participants highlighted language-specific, country-specific, and cultural-related challenges in taxonomy. The ranking of each statement within sections one to six is not presented as this was of limited value. Reasons stated were the complexity of obesity, with its multiple contributing factors, and the lack of clear weighing of these factors according to objective criteria.

**Table 3:** Comments from participants on specific statements.

Statement	Comments
**Definitions of obesity**	
Obesity is a complex disease	‘*I think the word "complex" has stagnated progress in policy and practice. It is almost as if practitioners and policymakers feel it is too challenging to address*’
Obesity is defined as abnormal or excessive adiposity that may impair, or present a risk to, health	*‘The term, may, needs to be removed. If there is no health impairment, there is no disease*’
	‘*I think the term "defined" is out of place. We don't need to define obesity just like we don't define diabetes. We never say*, "diabetes is defined". *We just say*, "Diabetes is a chronic, metabolic disease characterized by […]"’
Obesity is a chronic, complex disease defined by excessive adiposity that may impair health	Two participants highlighted that in German, obesity is synonymous with adiposity (adipositas) and that it could be confusing.
	Another participant was in favour of the simpler ‘fat accumulation’
	‘*I would like to have some reference to dysfunctional adipose tissue*’
	‘*Better adiposity than fat accumulation*’
Obesity can be measured by calculating BMI	*‘BMI is an epidemiological tool which does not define the disease of obesity’*
	*BMI is useful over large population sizes but might not be particularly useful on an individual basis'*
	*‘I would prefer EOSS’*
**Causes of obesity development**	
Psychological factors are among the main causes of developing obesity	Participants noted that these were all factually correct, but not one of the main causes of obesity
Pre-existing medical conditions are among the main causes of developing obesity	
Endocrine disorders are among the main causes of developing obesity	
Medication is among the main causes of developing obesity	
An imbalance between energy intake and energy output is among the main causes of developing obesity	‘*Energy imbalance is a result of something, so it is not the primary cause of obesity*’
	‘*This is more the result, not the cause in itself. The other factors may cause this imbalance*’
	‘*The imbalance is a symptom or a pathophysiologic mechanism, not the cause*’
**Factors exacerbating obesity**	
An imbalance between energy intake and energy output is among the main factors that exacerbate obesity	‘*Again, this is a consequence and not a cause*’
**Obesity treatment options**	
Obesity treatment options should aim at weight loss	*‘Weight loss is a consequence of successful treatment not an objective’*
	*‘Weight loss as such should not be an aim, especially not a number. Muscles are heavier than fat. If a person loses fat and gains muscles, that is a health improvement, and possible an increased weight’*
**Obesity management**	All statements on management received consensus among participants
**Medical complications of obesity**	
Type 2 diabetes is among the main medical complications of obesity	Participants noted that obesity could equally be a complication of diabetes

BMI: body mass index; EOSS: Edmonton Obesity Staging System.

## Discussion

Obesity is a heterogenous chronic relapsing disease^2^ that should be recognised and treated as such within the healthcare and policy sectors^23^; the results of this study reflect its complexity. The language used to counsel people living with obesity is paramount for achieving long-term health benefits.^6^ The use of appropriate language in the correct context is imperative in science and societal discussion in order to progress and deliver value for patients. Expert stakeholders consider that changes to language used at the point of care within the healthcare system can support improved outcomes both for people living with obesity and for the healthcare system.^24^ Standardised language is used to describe other chronic diseases covered by the 2011 United Nations political declaration on noncommunicable diseases.^25^ However, the language used to describe obesity is often inaccurate, which can contribute to discrimination, systemic bias, and the misunderstanding of obesity; these factors are key contributors to weight stigma.^23^ Healthcare professionals have a key role in ensuring that the conversations with people living with obesity are respectful, accurate, and precise. In line with other chronic diseases, there is a need for experts to have a shared understanding of the language, both context and usage, around obesity. The IMI-SOPHIA one voice study was a taxonomic classification exercise, conducted using the modified Delphi method. Use of this method was a strength of our study as it allows participants to express their viewpoints in a non-adversarial manner, acknowledges work already done in the area, and is designed to achieve group consensus.^13^ The aim of IMI-SOPHIA one voice was to map consensus across multiple stakeholders around the language used to discuss obesity as a chronic, relapsing disease. The present findings help clarify and propagate an equitable narrative around obesity to achieve a ‘one voice’ approach for the community. To the best of our knowledge at the time of writing, there is no study in the literature that has explored stakeholder consensus with a focus around the standard language used to discuss obesity as a chronic disease.

The results of our study showed consensus (≥70% agreement between experts) for the majority of statements that refer to obesity as a chronic, relapsing disease with heterogeneous aetiologies, as well as consensus around the inter-individual differences in the factors that affect disease progression, including genetic, epigenetic, psychological, and socio-economic factors. The results support a holistic approach to obesity treatment and management that is aligned with other complex, chronic diseases and show consensus on the broad range of diseases that can be considered complications of obesity.^26^^,^^27^

The design of our study allowed for the statements in Round 2 to be selected based on the responses of Round 1. As such, although not all statements in Round 2 achieved consensus, these were endorsed by some participants, highlighting the need for further discussion and granularity on these topics to achieve consensus. It is important to highlight that the findings of this study reflect the use of stakeholder language around obesity, and do not attempt to put into question the scientific mechanisms underlying obesity. As such, the lack of consensus regarding the use of some statements does not reflect the inaccuracy of these; rather that the use of these statements, or their context, was not agreed on by the participants. Consensus was not reached (<70% of expert agreement) for some commonly used language in obesity, including the use of statements that support energy imbalance as being among the main causes or exacerbating factors of obesity, and the use of BMI as a diagnostic biomarker for obesity. Although consensus was not reached for the statement pertaining to energy imbalance, this does not imply that there is no connection between energy intake/energy expenditure and obesity. The lack of consensus on the role of energy balance in obesity also does not exclude self-evident observations; for example, in some cases, the underlying disease can lead to increased responsiveness to food cues or increased feelings of hunger, resulting in an increase in energy intake. This indicates that the imbalance in energy intake and expenditure may be a consequence, rather than a cause, of the underlying pathophysiology. In other words, overeating may not cause obesity, but rather obesity may cause overeating. The lack of consensus regarding the statement ‘obesity can be defined by calculating BMI’ does not imply that BMI is not a relevant biomarker in the context of obesity; rather, participants indicated that BMI should not define obesity. This concept agrees with the notion that BMI is a highly useful epidemiological screening tool, but less useful at an individual level to make a clinical diagnosis of obesity. BMI can be used effectively to monitor the potential rates of obesity at the population level.^28^^,^^29^ This highlights the need to identify more meaningful clinical and biological criteria than just BMI to diagnose the disease.^23^^,^^30^ Consensus was also not reached regarding the idea that the only goal of obesity treatment should be weight loss; ‘weight’ is possibly too simplistic a biomarker when discussing treatment outcomes. The statements pertaining to treatment options taking a holistic approach with the aim of improving patient outcomes do not imply that weight management is not important, but rather that the treatment of people with obesity should have a broader focus, coupled with a multidisciplinary approach.

Our study had some limitations. It should be noted that English was not the first language for many of the experts. While this exercise was performed in English, vocabulary is translated differently into national languages, which can make achieving consensus more challenging. Translating the vocabulary into other European languages was beyond the scope of this study. Although the Expert Panel members and study participants were chosen to represent relevant stakeholder groups within and outside of Europe, the sample size was limited. Due to the small sample size, we could not analyse the difference in opinions by stakeholder category. Reviewing opinions by stakeholder category would be a worthy topic for future research. A select group of experts was enlisted, and therefore we cannot draw conclusions regarding the opinions of the stakeholders more broadly; however, further research on stakeholder opinion more widely is warranted to build on this study. Finally, due to the qualitative nature of our study, statistical analysis of results was not performed.

In conclusion, participants reached consensus on the majority of statements on the language of obesity included in this study, which focused on the complexity and heterogenous nature of the disease. The results also highlighted discrepancies compared with the established language of chronic diseases, and where consensus was still needed with respect to the language and context in some aspects of obesity. Taken together, this underlines the need for precise and scientifically accurate language of obesity, which can be used in communication tools to overcome the barriers and challenges that surround treatment and management of people living with obesity. By increasing awareness, partaking in collaborative discussions, and using appropriate language that considers the complexity of the disease, key stakeholders can play a role in improving communication around obesity across wider healthcare systems. The outcomes of this report are of relevance to inform researchers, policymakers, patient organisations, civil society, and industry.

## Contributors

Designed the study: CHJ, GHG, JBB.

Collected the data: CHJ, JBB, NA.

Interpreted the data: BAJ, CHJ, CIR, EG, JBB, NA.

All authors contributed to the drafting or critical revision of the manuscript.

All authors confirm full access to all the data in the study and accept responsibility to submit for publication.

JBB and CHJ have directly accessed and verified the underlying data reported in the manuscript.

## Data sharing statement

Data will be shared with bona fide researchers who submit a research proposal approved by an independent review board. Individual participant data will be shared in datasets in a de-identified and anonymised format. Data will be made available after research completion. Information about data access request proposals can be found at novonordisk-trials.com.

## Declaration of interests

CHJ is an employee of and shareholder at Novo Nordisk A/S.

JBB is contracted with EASO and Third-I (B2B) and is a volunteer with Belgian Patient Foundation.

TA is an employee of the European Federation of Pharmaceutical Industries and Associations.

NA is a non-paid Board Director of the European Association for the Study of Obesity (EASO) Early Career Network.

GHG received research funding from the Dutch Diabetes Research Foundation (DFN) and the European Foundation for the Study of Diabetes (EFSD) and is Co-Chair of the Scientific Advisory Board of the European Association for the Study of Obesity (EASO).

EG is a non-paid Deputy Chair of the ESDN Obesity of the European Federation of Associations of Dietitians (EFAD) and is a member of the Nutrition Working Group of EASO.

BAJ is an employee of Lilly Deutschland GmbH and shareholder at Eli Lilly.

VM is the Executive Director of the European Coalition for People Living with Obesity (ECPO) and Chairwoman of the Board of Irish Coalition for People Living with Obesity (ICPO).

BVDS is supported by a senior clinical research fellowship of the Flanders Research Foundation (1802719N).

CWlR reports grants from Anabio, the Health Research Board, the Irish Research Council, and Science Foundation Ireland. He serves on the advisory boards of Boehringer Ingelheim, Eli Lilly, GI Dynamics, Glia, Herbalife, Johnson & Johnson, and Novo Nordisk. CWlR is a member of the Irish Society for Nutrition and Metabolism outside the area of work commented on here. He was the Chief Medical Officer and Director of the Medical Device Division of Keyron in 2011. Both were unremunerated positions. CWlR was a previous investor in Keyron, which develops endoscopically implantable medical devices intended to mimic the surgical procedures of sleeve gastrectomy and gastric bypass. The product has only been tested in rodents and none of Keyron's products are currently licensed. They do not have any contracts with other companies to put their products into clinical practice. No patients have been included in any of Keyron's studies and they are not listed on the stock market. CWlR was gifted stock holdings in September 2021 and divested all stock holdings in Keyron in September 2021. He continues to provide scientific advice to Keyron for no remuneration.

## Appendix A Supplementary data

Supplementary Figures S1and S2
