# Supplementary material for: Achieving consensus on the language of obesity: a modified Delphi study
Source: eClinicalMedicine. 2023 Jul 27;62:102061. doi: 10.1016/j.eclinm.2023.102061 (PMC10404853; doi:10.1016/j.eclinm.2023.102061)
Supplement: Supplementary Figures S1and S2 [file mmc1.pdf]

Supplementary material

Table of contents

Figure S1. Top repeating terms and themes from respondent answers in Round 1 (questions 1, 2, and 3). 2

Figure S2. Top repeating terms and themes from respondent answers in Round 1 (question 4, 5, and 6)...3

Figure S1. Top repeating terms and themes from respondent answers in Round 1 (questions 1, 2, and 3).

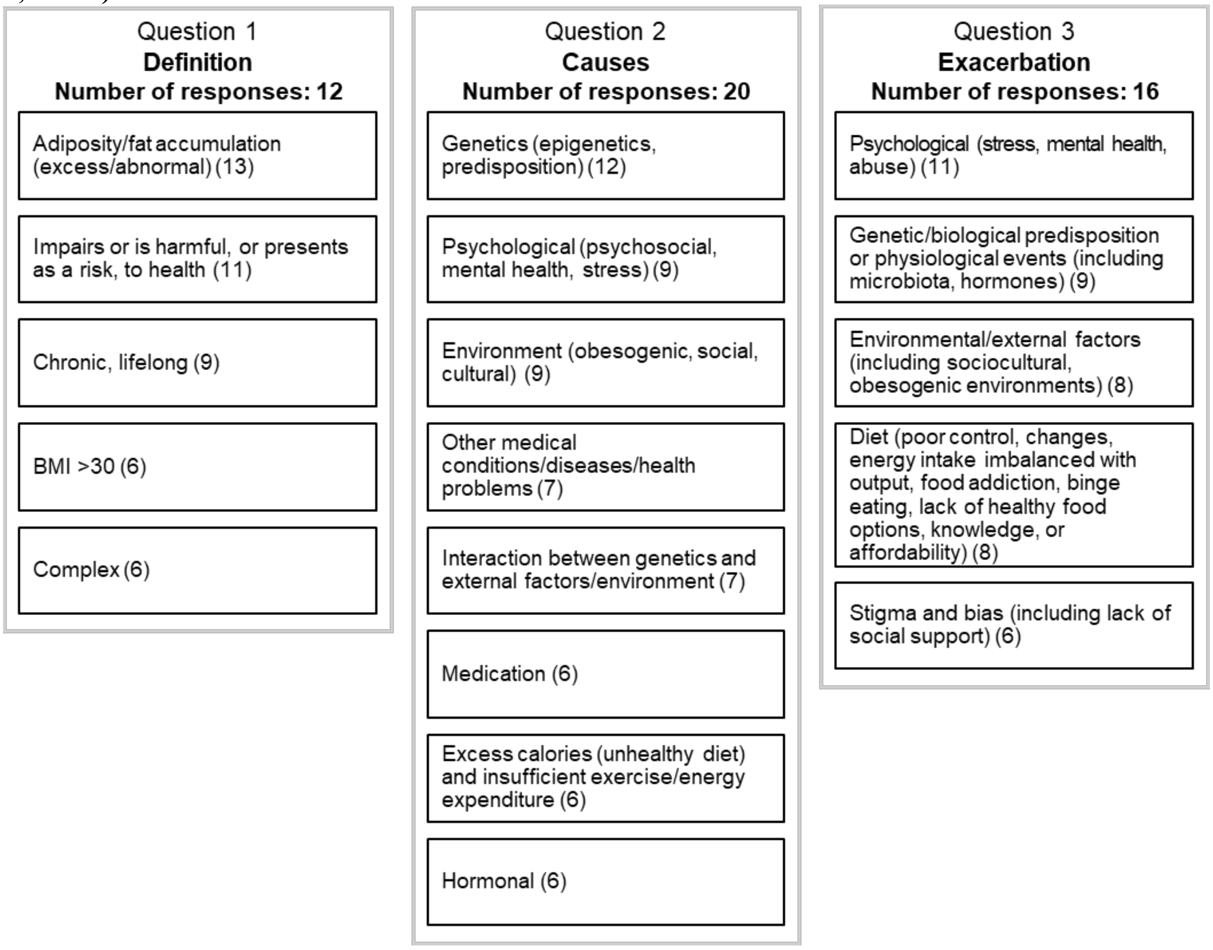

Numbers in brackets after statements indicate number of times of appearance.
BMI: body mass index.

Figure S2. Top repeating terms and themes from respondent answers in Round 1 (question 4, 5, and 6).

Question 4
Treatment
Number of responses: 20

- Bariatric surgery/surgery (16)
- Anti-obesity drugs/pharmacotherapy (15)
- Psychiatry, psychological support, cognitive behavioural therapy (13)
- Nutrition, meal plans and restrictive lifelong diets, eating habits (11)
- Multidisciplinary care/holistic approach (10)
- Physical activity (9)
- Any form of treatment that is prescribed to 'reduce weight' or more importantly to reduce fat and improve health outcomes, or maintain weight, or prevent weight re-gain (8)
- Lifestyle interventions (7)
- Patient-orientated care/ individualised (6)

Question 5
Management
Number of responses: 17

- Long-term approach, lifelong management (chronic, relapsing disease) (8)
- Personalised plan for a weight-reduction diet, increased physical activity and changes in behaviour and lifestyle, and other treatment if needed (4)
- Medical strategy put in place by the clinician together with the patient (4)
- All forms of obesity treatment, going beyond drugs or surgery, wider lifestyle changes, and education and other support (3)
- Improving overall health (healthy lifestyle and a better quality of life) not just weight loss (2)
- Weight loss and maintenance (2)
- Help from experts and loved ones, counselling and support programmes (2)

Question 6
Complications
Number of responses: 15

- Cardiovascular (stroke, hypertension, heart disease) (25)
- Type 2 diabetes/diabetes (17)
- Cancers (15)
- Psychosocial and psychological complications (mental health, depression, impact of stigma) (12)
- Arthrosis, osteoarticular, osteoarthritis, arthritis (5)
- Mechanical complications/physical function (5)

Numbers in brackets after statements indicate number of times of appearance.
